# Preventive Effects of Velvet Antler* (Cervus elaphus)* against Lipopolysaccharide-Induced Acute Lung Injury in Mice by Inhibiting MAPK/NF-*κ*B Activation and Inducing AMPK/Nrf2 Pathways

**DOI:** 10.1155/2018/2870503

**Published:** 2018-01-02

**Authors:** Jui-Shu Chang, Hung-Jen Lin, Jeng-Shyan Deng, Wen-Tzu Wu, Shyh-Shyun Huang, Guan-Jhong Huang

**Affiliations:** ^1^School of Chinese Medicine, Graduate Institute of Integrated Medicine, College of Chinese Medicine, China Medical University, Taichung, Taiwan; ^2^Chang Jui Shu Chinese Medicine Clinic, Changhua, Taiwan; ^3^Department of Health and Nutrition Biotechnology, Asia University, Taichung, Taiwan; ^4^School of Pharmacy, College of Pharmacy, China Medical University, Taichung 404, Taiwan; ^5^School of Chinese Pharmaceutical Sciences and Chinese Medicine Resources, College of Chinese Medicine, China Medical University, Taichung, Taiwan

## Abstract

Velvet antler (*Cervus elaphus*) is a typical traditional animal medicine. It is considered to have various pharmacological effects including stimulation of the immune system, increase in the physical strength, and enhancement of sexual function. This paper aims to investigate the aqueous extract of velvet antler (AVA) in the mouse models of LPS-induced ALI. Inhibition of NO, TNF-*α*, IL-1*β*, IL-6, and IL-10 productions contributes to the attenuation of LPS-induced lung inflammation by AVA. A 5-day pretreatment of AVA prevented histological alterations and enhanced antioxidant enzyme activity in lung tissues. AVA significantly reduced the material (total number of cells and proteins) in the BALF. Western blot analysis revealed that the expression of iNOS and COX-2 and phosphorylation of I*κ*B-*α* and MAPKs proteins are blocked in LPS-stimulated macrophages as well as LPS-induced lung injury in mice. Consistent with this concept, the phosphorylation of CaMKK*β*, LKB1, AMPK, Nrf2, and HO-1 was activated after AVA treatment. The results from this study indicate AVA has anti-inflammatory effects in vivo and AVA is a potential model for the development of health food. In addition, its pathways may be at least partially associated with inhibiting MAPK/NF-*κ*B activation and upregulating AMPK/Nrf2 pathways and the regulation of antioxidant enzyme activity.

## 1. Introduction

Acute lung injury (ALI) is the most common form of respiratory failure, which is shown by increasing capillary-alveolar permeability, changing in lung protein leaks, inflammatory cell accumulation, interstitial edema, disruption of the alveolar epithelium, excessive polymorphonuclear neutrophil (PMN) migration, and the release of proinflammatory cytokines and mediators in the lung [1]. This disease is associated with a great risk of morbidity and mortality in patients with shock, sepsis, ischemia-reperfusion, aspiration of gastric contents, major trauma, and other clinical disorders [2]. Macrophages can be activated by cytokines such as interferon-*γ* (IFN-*γ*) and lipopolysaccharide (LPS, bacterial endotoxin) and downregulation of proinflammatory cytokine release [tumor necrosis factor-alpha (TNF-*α*), interleukin-1*β* (IL-1*β*), and IL-6] and inflammatory factors (NO) that recruit additional immune cells to the tissue injury [3, 4]. Also, LPS promotes the production of reactive oxygen species (ROS) and reactive nitrogen species (RNS). NO induced after LPS challenge reacts with O^2−^ and produces peroxynitrite (ONOO−). ONOO− is linked to cell death and lung injury. Thus, the excessive production of ROS and RNS may suppress the innate defense [5, 6]. However, the mechanism of neutrophil migration and pathogenesis in LPS-induced ALI is not completely understood, though previous studies have shown that the participation of nuclear factor-*κ*B (NF-*κ*B) and mitogen-activated protein kinase (MAPK) pathways are related to the pathogenesis of the syndrome [7].

AMP-activated protein kinase (AMPK) is a key moderator in inflammatory processes and also plays a crucial role in the regulation of cellular energy homeostasis. AMPK activation occurs through phosphorylation at threonine-172 (Thr-172) of the *α*-subunit by liver kinase B1 (LKB1) or calcium/calmodulin-dependent protein kinase kinase *β* (CaMKK*β*) in response to energy deprivation or an increase in intracellular Ca^2+^, respectively [8]. Experiments have demonstrated that activation of AMPK could be inhibited by the LPS-induced inflammatory response in vitro [9]. Under normal conditions, cytoplasmic nuclear factor erythroid-2-related factor (Nrf2) is regulated via its interaction with Kelch-like ECH-associated protein (Keap1). Oxidative stress or stimulation of nucleophilic substances may trigger dissociation of Nrf2 from Keap1 and release free Nrf2. Following disconnection, Nrf2 is translocated from the cytoplasm to the nucleus and activates the promoter regions of antioxidant response element (ARE), triggering not only downstream detoxifying enzymes such as HO-1 but also anti-inflammatory mechanisms associated with NF-*κ*B-mediated inflammatory responses accompanying iNOS, COX-2 enzyme expression, cytokine release, and NO production [10]. Although activation of AMPK has recently been reported, little information has been obtained on how AMPK/Nrf2 may play an important role in modulating functions in the inflammatory response, such as acute lung injury.

Velvet antler* (Cervus elaphus)* has been used as a medicinal herb and functional food in China for thousands of years as an aid to help people maintain overall wellness. Nowadays, velvet antler is recognized as a traditional medicine in pharmacopeias of China, Korea, Japan, and Taiwan. Velvet antler also has been reported to have pharmacological activity, such as immunomodulatory [11], anti-inflammatory [12], antiaging [13], wound-healing [14], and anticancer [15] effects. The present study investigates the aqueous extract of velvet antler (AVA) that ameliorates LPS-induced acute lung injury in mice. Our results suggest that AVA is a good candidate for the development of dietary supplements to prevent acute lung injury and to inhibit inflammation.

## 2. Materials and Methods

### 2.1. Materials and Reagents

Frozen deer velvet* (Cervus elaphus)* from New Zealand elks was provided from KO DA Pharmaceutical Co., Ltd. (Taoyuan, Taiwan). LPS (endotoxin from* Escherichia coli*, serotype O127:B8), dexamethasone (Dex), and other chemicals were all of analytical grade from Sigma-Aldrich, Inc. (St. Louis, MO). TNF-*α*, IL-1*β*, IL-6, and IL-10 enzyme-linked immunosorbent assay (ELISA) kits were supplied by BioSource International Inc. (Camarillo, CA, USA). All antibodies were purchased from Cell Signaling Technology (Beverly, MA, USA).

### 2.2. Antler Extract

Dry powder of velvet antler (100 grams) was extracted separately in 500 mL of boiling water for one hour. The sample was reextracted 3 times. The extract obtained was concentrated under vacuum at 45°C and freeze-dried for 24 h. All freeze-dried extracts were kept at 4°C prior to analyses.

### 2.3. Animals

Animal studies were conducted according to the NIH* Guide for the Care and Use of Laboratory Animals*, and all tests were conducted under the guidelines of the International Association for the Study of Pain. Six-week-old male ICR (Imprinting Control Region) mice (25–32 g) were randomly assigned to six groups (*n* = 6). The control group received normal saline (intraperitoneal, i.p.). The other groups included an LPS-treated group (5 mg/kg), a positive control group (LPS + Dex, 10 mg/kg), and a group that was given AVA (LPS + 125 mg/kg AVA, LPS + 250 mg/kg AVA, and LPS + 500 mg/kg AVA).

### 2.4. LPS-Induced Acute Lung Injury in Mice

LPS-induced ALI in ICR mice was performed as described in previous studies [16]. Mice were randomly assigned to six groups (*n* = 6 each): a sham operation group and five treatment groups. Mice were intratracheally given 5 mg/kg LPS to induce lung injury, while sterile saline was used as the control. AVA was ground and suspended in distilled water for administration to the mice. The AVA-pretreated groups received an intragastric injection of AVA at the indicated doses, and each mouse was given the drug orally once per day for 5 consecutive days. The positive group received dexamethasone (Dex; 10 mg/kg) only on day 5. Mice from the control and positive groups received an equal volume of distilled water instead of AVA. The doses of these drugs were chosen on the basis of our previous studies and preliminary experiments.

On day 5, 1 h after AVA or Dex treatments, mice were slightly anesthetized intraperitoneally with a mixed reagent of 10 *μ*L/g i.p. urethane (0.6 g/mL) and chloral hydrate (0.4 g/mL). Then, in the positive and experimental groups, 5 mg/kg LPS was instilled intratracheally (i.t.) in 50 *μ*L of sterile saline to induce lung injury or sterile saline alone (control group). It took approximately 2-3 min per mouse to induce lung injury. The animals recovered quickly from the procedure with only mild discomfort. The mice were sacrificed 6 h after the LPS challenge, and blood samples were collected. The right lung was used to collect bronchoalveolar lavage fluid (BALF), which was lavaged three times with 0.6 mL of autoclaved sterile saline and evaluated histopathologically.

The BALF was collected for TNF-*α*, IL-1*β*, IL-6, IL-10, and protein concentration levels and leukocyte infiltration assessment and then centrifuged at the speed of 700 ×g for 10 min at 4°C, and their supernatants were frozen at −80°C. The sediment cells were resuspended in 2 mL PBS; half of them were used to detect cell counts by a cytometer; the rest were equally divided into two parts. One was centrifuged again in order to get a sediment for extracting proteins with a RIPA solution (radioimmunoprecipitation assay buffer) and centrifuged again to obtain the supernatant in order to detect total protein content by Bradford assay.

### 2.5. Histopathological Analysis

The right lungs were harvested after treatment with LPS at 6 h and fixed in 4% paraformaldehyde in 0.1 M PBS (pH 7.4) buffer for 24 h. The lung tissue was dehydrated and embedded in paraffin before being stained with hematoxylin and eosin (H&E) and observed under a light microscope. The score represented severity of lung injury which depended on the degree of inflammatory cell infiltration: neutrophilic, alveolus, and diffuse. Based on the severity of lesions, the score was graded from one to five. A score of 0 represented no abnormalities; 1 represented minimal (<1%); 2 represented slight (1–25%); 3 represented moderate (26–50%); 4 represented moderate/severe (51–75%); 5 represented severe/high (76–100%) [16].

### 2.6. Measurement of Nitric Oxide/Nitrite

NO production was indirectly assessed by a colorimetric method based on the Griess reaction in the BALF [17]. The Griess reagent was prepared by mixing together equal volumes of 0.1% N-1-naphthylethylenediamine dihydrochloride and 1% sulfanilamide. The optical density (OD) of each well was assayed at 540 nm with a microplate spectrophotometer. Finally, the contents were calculated according to the concentration of sodium nitrite.

### 2.7. Lung Wet-to-Dry Weight (W/D) Ratio

After LPS administration, the mice were euthanized at 6 h, and the right lung tissues were excised to obtain the wet weight and then placed in an oven at 65°C for 72 h to obtain the dry weight. The W/D ratio was then calculated to assess tissue edema.

### 2.8. Measurement of Myeloperoxidase (MPO) Activity

MPO activity reflects the parenchymal infiltration of neutrophils and macrophages [18]. The lungs were homogenized in 50 mM potassium phosphate buffer (pH 6.0) and suspended in 50 mM potassium phosphate buffer containing 0.19 mg/mL* o*-dianisidine chloride and 0.005% H_2_O_2_ as a substrate. Oxidized* o*-dianisidine was determined for myeloperoxidase at 460 nm by spectrophotometry. MPO content was expressed as relative MPO activity (OD 460 nm/mg protein of lung tissue).

### 2.9. Measurement of BALF TNF-*α*, IL-1*β*, IL-6, and IL-10 by Enzyme-Linked Immunosorbent Assay (ELISA)

Analysis was performed using commercially available ELISA kits (BioSource International Inc., Camarillo, CA, USA) for TNF-*α*, IL-1*β*, IL-6, and IL-10 cytokines in the BALF according to the manufacturer's instructions. TNF-*α*, IL-1*β*, IL-6, and IL-10 were determined using a standard curve.

### 2.10. Measurement of the Levels of ROS Production in BALF

The effect of AVA on ROS generation was determined by DCFH-DA (2′,7′-dichlorodihydrofluorescein diacetate). Briefly, the cells in BALF were washed with PBS and incubated with 100 *μ*M DCF-DA (2′,7′-dichlorofluorescein diacetate) in the dark for 30 minutes at 37°C. DCF (2′-7′-dichlorofluorescein) fluorescence intensity was measured by Synergy HT Microplate Reader (BioTek Instruments) with an excitation wavelength of 485 nm and emission wavelength of 535 nm.

### 2.11. Western Blot Analysis

Lung tissues were homogenized and the proteins were extracted in tissue lysis buffer. The supernatants was then separated on a denaturing 10% SDS-polyacrylamide gel and transferred to polyvinylidene difluoride membranes. The membrane was blocked for 2 h with 5% skim milk in Tris buffered saline (TBS; 20 mM Tris and 500 mM NaCl, pH 7.5) followed by incubation with mouse monoclonal antibodies against iNOS, COX-2, I*κ*B-*α*, NF-*κ*B (p65), ERK, JNK, p38, p-ERK, p-JNK, p-p38, SOD, catalase, GPx, p-CaMKK*β*, p-LKB1, p-AMPK, AMPK, HO-1, Nrf2, Keap1, and *β*-actin antibodies overnight. The secondary antibody (anti-mouse IgG secondary antibody conjugated to horseradish peroxidase, 1 : 2000) was incubated in 2.5% skim milk in TBS for 1 h. Detection was performed using the enhanced chemiluminescence system (Amersham International Plc., Buckinghamshire, UK). The results were quantified by measuring the relative intensity compared to the control using Kodak Molecular Imaging Software (Version 4.0.5, Eastman Kodak Company, Rochester, NY, USA) and are represented as relative intensities.

### 2.12. Statistical Analysis

Experimental results were presented as the mean standard error (±SD). Statistical evaluation was determined by one-way analysis of variance (ANOVA) followed by Scheffe's multiple range test. Statistical significance is expressed as ^*∗*^ *p* < 0.05, ^*∗∗*^ *p* < 0.01, and ^*∗∗∗*^ *p* < 0.001.

## 3. Results

### 3.1. AVA Reduces LPS-Induced Murine Lung Injury In Vivo

**Figure 1:**
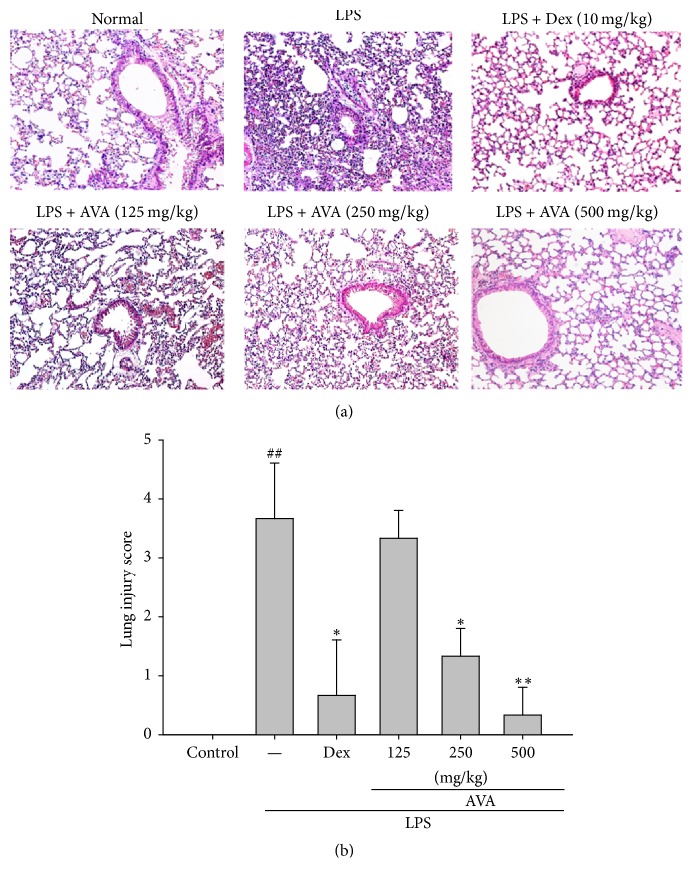
The effects of the aqueous extract of velvet antler (AVA) on histopathological changes in the lung (a) and on the severity of lung injury were analyzed using the lung injury scoring system (b) in LPS-induced ALI mice. Mice were sacrificed 6 h after LPS stimulation. The right lungs were excised and embedded in 10% formalin, sectioned, and stained with H&E (magnification ×400). Images are representative of three experiments. Dex: dexamethasone. The data are presented as the mean ± SD for three different experiments performed in triplicate. ^##^Compared with the control group. ^*∗*^ *p* < 0.05 and ^*∗∗*^ *p* < 0.01 compared with the LPS-alone group.

In an experimental study to assess the effect of AVA on LPS-induced ALI, we noticed the imaging features of different histologic subtypes of lung tissues. The normal group showed a normal structure in lung specimens (Figure 1(a)). The acute LPS-induced pulmonary injury group with H&E staining exhibited significant histopathological changes such as a large amount of neutrophil infiltration, alveolar walls edema, and alveolar hemorrhage. These pathological changes were improved by various concentrations of AVA (125, 250, or 500 mg/mL) or Dex (10 mg/kg), suggesting that AVA could prevent the histological changes in the LPS-induced animal models. AVA dose-dependently decreased LPS-induced lung edema, as revealed by the pulmonary pathological scores (Figure 1(b)). These findings suggest that AVA protected against LPS-induced histological changes in the ALI model.

### 3.2. AVA Attenuates Increased Protein Concentrations and Total Cell Count in LPS-Induced ALI Mice

**Figure 2:**
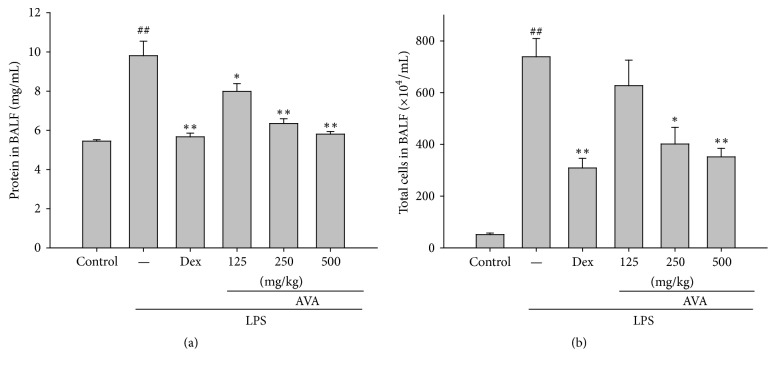
Effects of AVA on the total protein level (a) and total cell number (b) in the BALF of LPS-induced ALI mice. Mice were given AVA (125, 250, and 500 mg/kg) by intraperitoneal injection 1 h before challenge with LPS. The BALF was collected 6 h after LPS challenge. Cell counts were assessed using a hemocytometer. Each value represents the mean ± SD. ^##^ *p* < 0.01 compared with the control group. ^*∗*^ *p* < 0.05 and ^*∗∗*^ *p* < 0.01 compared with the LPS group (one-way ANOVA followed by Scheffe's multiple range test).

We investigated protein concentrations in different groups in LPS-induced ALI mice.  Figure 2(a) shows that protein concentrations increased significantly in mice in the LPS group. Pretreatment with AVA (125, 250, or 500 mg/mL) and Dex (10 mg/kg) decreased protein concentrations remarkably. These results indicate that AVA alleviated pulmonary edema in LPS-induced ALI mice.

The total number of cells increased markedly after the intratracheal instillation of LPS after 6 h compared to the control group. The number of total cells in AVA- (250 or 500 mg/mL) and Dex- (10 mg/kg) treated mice also significantly decreased compared to the LPS-induced ALI mice (Figure 2(b)). These results evidence that AVA reduced excessive neutrophils activation in the lung tissues and inhibited lung injury in LPS-induced inflammation.

### 3.3. Effects of the W/D Ratio in LPS-Induced Acute Lung Injury

**Figure 3:**
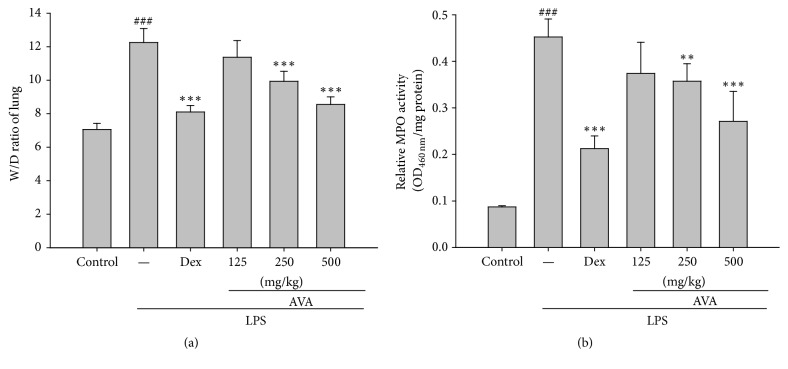
The effects of AVA on the lung W/D ratio (a) and myeloperoxidase activity (b) in LPS-induced ALI mice. Mice were given sclareol 1 h prior to intratracheal (i.t.) administration of LPS. The lung W/D ratio (a) and myeloperoxidase activity (b) were determined 6 h after LPS challenge. Each value represents the mean ± SD. ^###^ *p* < 0.001 compared with the control group. ^*∗∗*^ *p* < 0.01 and ^*∗∗∗*^ *p* < 0.001 compared with the LPS group (one-way ANOVA followed by Scheffe's multiple range test).

For the examination of lung edema, we measured the water content after 6 h in LPS-induced ALI mice. LPS-induced ALI increases the pulmonary vascular permeability as shown by the lung wet/dry weight (W/D) ratio compared to the uninjured control group. In Figure 3(a), the W/D ratio increased in the LPS-induced group and the pretreatment with AVA (250 and 500 mg/kg) or Dex ameliorated the W/D ratio compared with the LPS-induced group. These results indicated that AVA attenuates LPS-induced ALI in mice by alleviating lung edema.

### 3.4. Effects of AVA on MPO Activity in LPS-Induced ALI in Mice

PMN infiltration is known to play a major role in inflammatory reaction, and it is also the most common cause of tissue damage. MPO activity could be a key indicator of lipid peroxidation in lung tissues. As Figure 3(b) displays, mice that were intratracheally given LPS had significantly increased MPO activity in lung tissues. Pretreatment with AVA (250 or 500 mg/kg) or Dex (10 mg/kg) decreased MPO activity. These data suggested that AVA prevents excessive release of macrophages and neutrophil-derived inflammation in LPS-induced ALI mice.

### 3.5. Effect of AVA on the Production of Proinflammatory Cytokines in Lung Tissues of LPS-Induced ALI Mice

**Figure 4:**
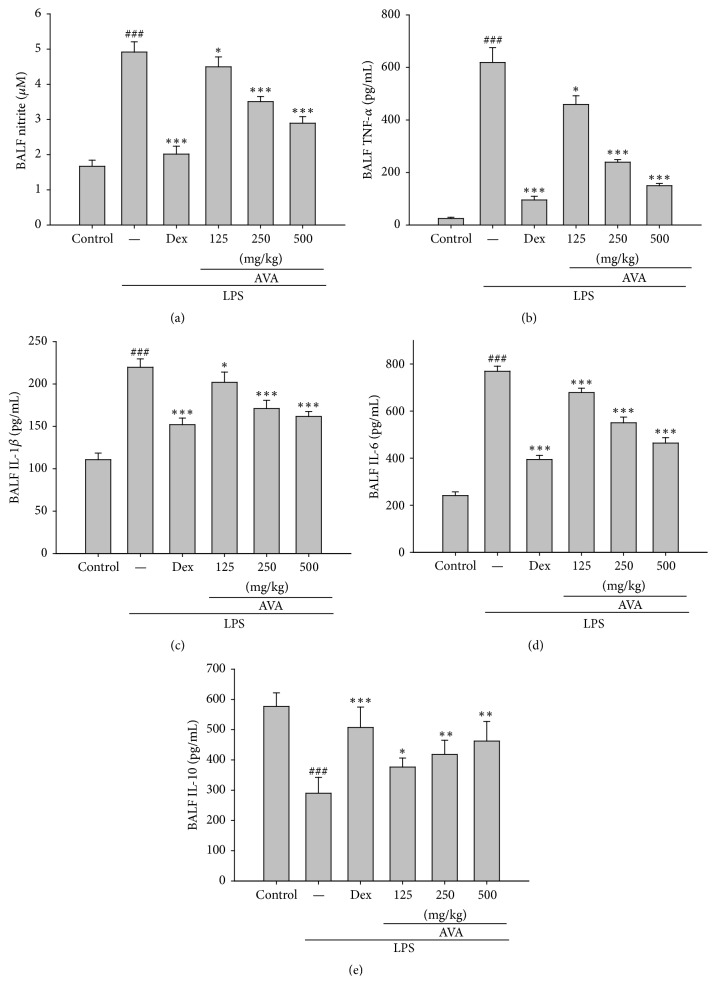
The effect of AVA on NO (a), TNF-*α* (b), IL-1*β* (c), IL-6 (d), and IL-10 (f) expression in mice with ALI. Mice were given AVA (125, 250, and 500 mg/kg) via intraperitoneal injection 1 h before challenge with LPS. The BALF was collected 6 h following LPS challenge to analyze the inflammatory cytokines NO, TNF-*α*, IL-1*β*, IL-6, and IL-10. Each value represents the mean ± SD. ^###^ *p* < 0.001 compared with the control group. ^*∗*^ *p* < 0.05, ^*∗∗*^ *p* < 0.01, and ^*∗∗∗*^ *p* < 0.001 compared with the LPS group (one-way ANOVA followed by Scheffe's multiple range test).

To evaluate the levels of NO, TNF-*α*, IL-1*β*, IL-6, and IL-10 in BALF, BALF was collected 6 h after LPS administration. NO, TNF-*α*, IL-1*β*, IL-6, and IL-10 levels in the BALF of LPS-induced ALI mice were significantly increased compared with the control group (Figures 4(a), 4(b), 4(c), 4(d), and 4(e)). AVA (125, 250, or 500 mg/kg) or Dex (10 mg/kg) significantly reduced NO, TNF-*α*, IL-1*β*, and IL-6 and elevated IL-10 production compared to the LPS-induced ALI group.

### 3.6. AVA Protects against Oxidative Stress in LPS-Induced ALI Mice

**Figure 5:**
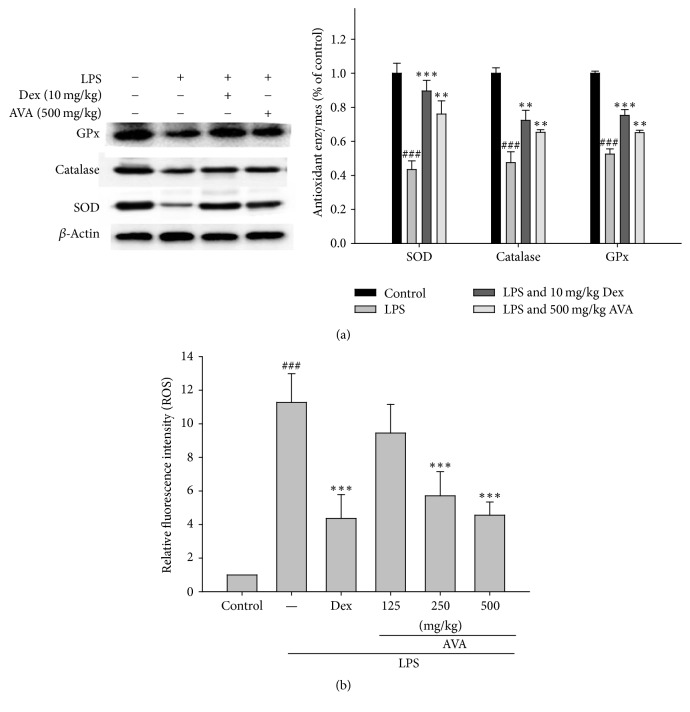
The antioxidant enzyme activity (a) and ROS production (b) of AVA in lungs from mice with ALI. Tissue homogenates were prepared and subjected to western blotting using antibodies specific for SOD, catalase, and GPx. *β*-Actin was used as an internal control. The BALF was collected 6 h following LPS challenge to analyze ROS production. The data are presented as the mean ± SD for three different experiments performed in triplicate. ^###^Compared with the control group. ^*∗∗*^ *p* < 0.01 and ^*∗∗∗*^ *p* < 0.001 compared with the LPS-alone group.

Oxidative stress appears to play a role in regulating inflammatory status in the lungs [19]. The presented data demonstrated that the pretreatment with AVA (500 mg/kg) or Dex increased the antioxidant enzymes activity (catalase, SOD, and GPx) in mice compared to the LPS-induced ALI group (Figure 5(a)). Therefore, the effects of AVA might be increased to its antioxidant enzyme activities in the LPS-induced ALI mice. In addition, we investigated whether AVA attenuates LPS-stimulated ROS production. As shown in Figure 5(b), AVA treatment significantly reduced ROS production in a concentration-dependent manner in LPS-induced ALI mice. These findings suggest that the anti-inflammatory effect of AVA may be associated with its antioxidant effects in LPS-induced ALI mice.

### 3.7. Effects of AVA on LPS-Induced iNOS and COX-2 Protein Expression in ALI Mouse Lung Tissues

**Figure 6:**
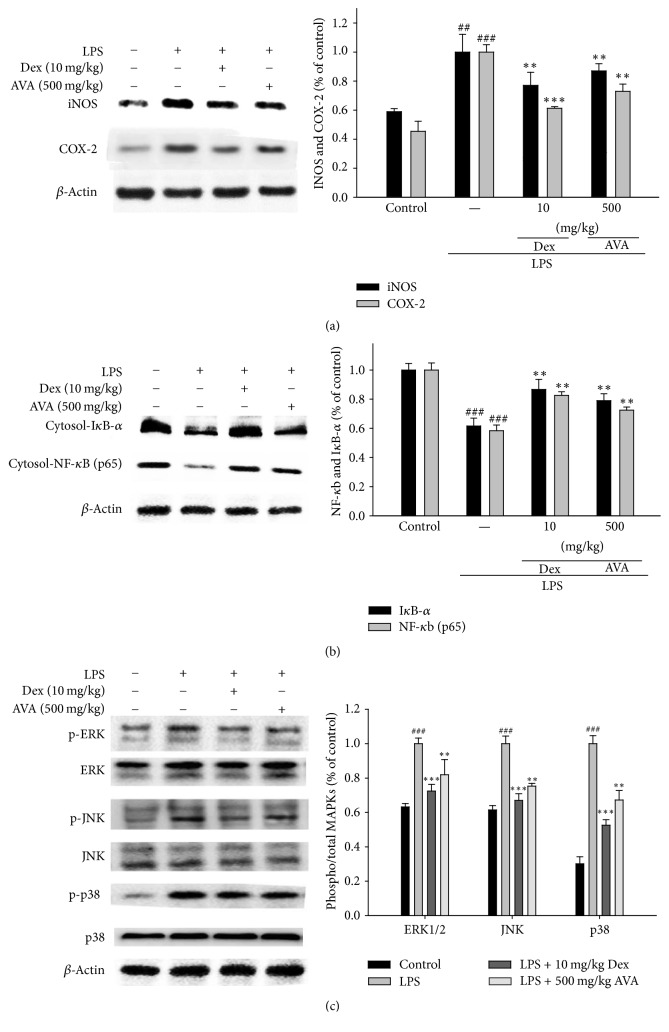
The inhibition of iNOS, COX-2 (a), I*κ*B-*α*, NF-*κ*B (b), and MAPK protein (c) expression by AVA in LPS-induced ALI mice. Tissue homogenates were prepared and subjected to western blotting using antibodies specific for iNOS, COX-2, I*κ*B-*α*, NF-*κ*B, and MAPK. The values under each lane indicate the relative band intensities normalized to *β*-actin. The data are presented as the mean ± SD for three different experiments performed in triplicate. ^###^Compared with the control group. ^*∗∗*^ *p* < 0.01 and ^*∗∗∗*^ *p* < 0.001 compared with the LPS-alone group.

Mouse iNOS and COX-2 protein expression levels were studied with pretreatment of AVA in LPS-induced ALI using western blots. The results evidenced that the pretreatment with AVA (500 mg/kg) inhibited iNOS and COX-2 protein expression levels in the LPS-induced ALI mice (Figure 6(a)). *β*-Actin was used as an internal control.

### 3.8. Effect of AVA on NF-*κ*B and I*κ*B-*α* Inactivation in LPS-Induced ALI Mice

NF-*κ*B can be activated by many stimuli, such as LPS, TNF-*α*, and IL-1*β*, and is involved in biological responses [17]. In our study, Figure 6(b) shows that the expressions of I*κ*B-*α* and NF-*κ*B were significantly inhibited by AVA in the LPS-induced ALI mice. The NF-*κ*B signaling pathway suppressed the export of nuclear NF-*κ*B by inhibition of I*κ*B*α*.

### 3.9. Effect of AVA on the MAPK Signaling Pathway in LPS-Induced ALI Mice

We checked the effect of AVA on the MAPKs signaling pathway in the LPS-induced ALI mice. The results are shown in Figure 6(c); the level of expression of phosphate MAPKs was enhanced in LPS-induced ALI. Further, AVA (500 mg/kg) or Dex dramatically decreased the expression of phosphorylation of ERK, JNK, and p38 protein levels. These results revealed that AVA inhibited the activity of MAPK pathways in LPS-induced ALI.

### 3.10. Effect of AVA on the AMPK and Nrf2 Signaling Pathway in LPS-Induced ALI Mice

**Figure 7:**
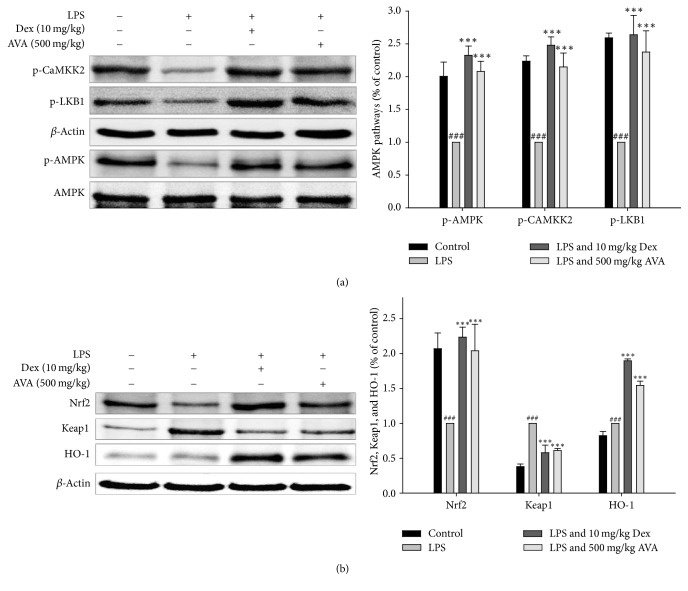
The inhibition of CaMKK*β*, LKB1, and AMPK (a) and HO-1, Nrf2, and Keap1 (b) expression by AVA in LPS-induced ALI mice. Tissue homogenates were prepared and subjected to western blotting using antibodies specific for p-CaMKK*β*, p-LKB1, p-AMPK, AMPK, HO-1, Nrf2, and Keap1. The values under each lane indicate the relative band intensities normalized to *β*-actin. The data are presented as the mean ± SD for three different experiments performed in triplicate. ^###^Compared with the control group. ^*∗∗∗*^ *p* < 0.001 compared with the LPS-alone group.

We investigated the AMPK and Nrf2 signaling pathways in LPS-induced ALI mice. As shown in Figure 7(a), the phosphorylation of LKB1, CaMKK*β*, and AMPK was elevated after AVA (500 mg/kg) treatment in LPS-induced ALI. Further, AVA or Dex dramatically increased the Nrf2 and HO-1 protein expression levels and decreased the Keap1 protein expression level in the cytoplasm (Figure 7(b)). These results demonstrated that AVA elevated the activity of AMPK/Nrf2 pathways in LPS-induced ALI.

## 4. Discussion

Traditional animal medicine has long been used for the treatment of diverse diseases. Velvet antler can reduce aching, dry throat, restlessness, dizziness, tinnitus, memory loss, emaciation, night sweats, hair loss, and scanty urine with swollen ankles. In this study, for the first time, with the anti-inflammatory properties of AVA, we showed that AVA ameliorates LPS-induced acute lung injury in mice. We found that pretreatment with AVA reduced LPS-induced pulmonary edema, proinflammatory cytokine production in the BALF, MPO activity, and the phosphorylation of NF-*κ*B/P65, I*κ*B-*α*, and MAPKs signaling pathways. All the results of this investigation demonstrated that AVA might have several additional anti-inflammatory effects in LPS-induced ALI. ALI is characterized by damage to the alveolar-capillary barrier and increased lung tissue permeability, thereby resulting in pulmonary edema, the infiltration of neutrophils into the alveolar space, and hypoxia, which finally affect breathing function [20].

ALI is a serious clinical problem in Taiwan with high mortality in shock, ventilator use, hypoxia, sepsis, and ischemia-reperfusion. LPS-induced ALI has been used for a long time to study the biological pathways of ALI [21]. Therefore, intratracheal LPS-induced ALI model is well established for the induction of localized inflammation in vivo and is well suited to the study of potential preliminary preventive or therapeutic compounds against ALI. Activation of neutrophils plays a key role in regulating LPS-induced ALI. Neutrophils were induced to migrate across the endothelium into the alveolar space, which causes the release of numerous ROS and proinflammatory mediators [22]. The result is a cascading response to injury. Proinflammatory cytokines, like TNF-*α*, IL-1*β*, and IL-6, by neutrophils are produced, which have close links with respiratory diseases. Finally, neutrophil infiltration leads to hypoxia and pulmonary edema associated with hyaline membrane formation in the alveolar walls [23]. In this study, we carefully showed the effect of AVA in the LPS-induced ALI model by intratracheal instillation with the aim of establishing the potential of the compound for use in ALI treatment. At present, we found that AVA improved the LPS-induced histopathological changes, such as infiltration of neutrophils, hemorrhage, and alveolar edema in the lung tissues. These results suggested that AVA can be considered as a valuable preventive agent for ALI.

MPO activity is a sensitive and specific marker for acute lung injury that is used to assess the quantification of neutrophil accumulation in tissues [24]. The present study was carried out to evaluate whether the AVA treatment significantly reduced the number of total cells in BALF and the MPO activity in lung tissues compared with the LPS group. MPO activity, caused by the most abundant enzymes in the neutrophil's granules, has been related to many inflammatory diseases. MPO activity is an important index of neutrophil infiltration into lung parenchyma or alveolar spaces in ALI [25].

In ALI or sepsis patients, proinflammatory cytokines persistent elevation always predicts a worse outcome. Both TNF-*α* and IL-1*β* increase the permeability of vascular endothelial cells and furthermore induce alveolar epithelial cells to produce other interleukins [26]. IL-6 is also increased in the BALF of patients and higher levels increase mortality. In addition, IL-10 has been reported to be a potent immunomodulatory cytokine in counterbalancing the proinflammatory response with diverse cellular production. It was previously observed that the IL-10 levels increase in ALI mice [27]. These results suggest that the suppression of inflammation by AVA in the lung is associated with its inhibition of proinflammatory cytokine release.

ROS plays an important role in the development of LPS-induced acute lung injury, in which the excessive production of ROS leads to an imbalance of the antioxidant system and causes lung tissue damage. Antioxidant activities are mediated by antioxidant enzymes such as SOD, catalase, and GPx. These enzymes stimulate the repair of cells damage caused by the accumulation of ROS [28]. In the present study, we found that pretreatment with AVA prior to LPS injection increased the activity of SOD, catalase, and GPx in the lungs of mice.

LPS was found to be a potent stimulator of iNOS expression, which further causes the overproduction of NO. The role of excessive NO production plays an important role by inducing tissue dysfunction and ONOO− formation. Inhibition of ROS, NO, and ONOO− is the major contributing factor to alleviating acute lung injury [29]. In our experiment, the levels of iNOS expression and NO in the lung were significantly reduced by AVA.

NF-*κ*B is a pleiotropic transcription factor which is present in almost all cell types by the inhibitor protein I*κ*B-*α*. Activation by LPS leads to the release of NF-*κ*Bp65. Subsequently, NF-*κ*B translocates to the nucleus, binds to DNA, and promotes the transcription of responsive genes in the nucleus, such as TNF-*α* and IL-6 [30]. NF-*κ*B is a novel type transcription factor involved in inflammation, apoptosis, and proliferation [31]. In the present study, it is evidenced that pretreatment with AVA inhibits the phosphorylation of I*κ*B-*α* and prevents its nuclear translocation. AVA markedly suppressed NF-*κ*B activation through the inhibition of I*κ*B-*α* degradation. TNF-*α* and IL-6 were believed to be of importance to the inflammatory response in ALI [32]. The elevated TNF-*α* and IL-6 protein expressions were significantly lowered with the administration of AVA.

The MAPKs pathway (p38, ERK, and JNK) acts as a cellular behavior in response to extracellular stimuli that control the synthesis and release of proinflammatory mediators by activating macrophage-mediated inflammatory responses [33]. In this study, pretreatment with AVA suppressed LPS-induced phosphorylation of the MAPKs pathway (p38, ERK, and JNK). The inhibition of TNF-*α*, IL-1*β*, and IL-6 production by AVA might occur through pathways that converge on NF-*κ*B activation because they can regulate cytokine production in LPS-induced acute lung injury. In this study, we found that AVA significantly inhibited LPS-induced I*κ*B-*α* degradation and phosphorylation of NF-*κ*B and MAPK.

Nrf2 plays a key role in various inflammatory reactions and oxidative stress-induced diseases, such as ALI [34]. Under unstressed conditions, Nrf2 is bound to Keap1 in the cytoplasm. Upon exposure to stressors and inducers, Nrf2 is released from Keap1 and translocates into the nucleus of cytoprotective genes affecting several protective systems, such as HO-1, dependent upon binding to conserved AREs [35]. In addition, AMPK pathway plays a key role in regulating many important proteins involved in the anti-inflammatory response, by inducing HO-1 and Nrf2 actions [16]. The phosphorylation of AMPK was moderately enhanced by treatment with AVA. These results suggest that AVA activates the AMPK signaling pathway and mediates the HO-1 and Nrf2 protein expressions in LPS-induced lung injury. Given the association of AVA with the AMPK pathway, we explored the AVA-dependent role of AMPK anti-inflammation in this study. We have proved that the AVA-dependent activation of AMPK inhibited iNOS, COX-2, and NF-kB promoter activity against LPS-induced ALI.

Velvet antler refers to the whole cartilaginous antler in a precalcified stage. It was used as a tonic in traditional Chinese medicine for thousands of years as an aid to help people maintain overall wellness. In scientific papers, it was evidenced that velvet antler alcalase hydrolysate (VAAH) inhibited NO, iNOS, and COX2 production in inflammatory cells and it contained bioactive components such as sialic acid and uronic acid [36]. Furthermore, VAAH exhibited a stronger anti-inflammatory action and also exhibited a potent anti-inflammatory effect in a zebrafish model. In addition, the anti-inflammatory peptides from simulated gastrointestinal digest of velvet antler protein suppress NO production in LPS-induced RAW264.7 cells [15, 37]. Although velvet antler active substances for therapeutic effects have not been identified, several studies have reported the presence of complex lipids, such as glycolipids and phospholipids, as the active substances. In this study, there was a significant improvement in iNOS, COX-2, NF-*κ*B, and MAPK activities and inflammatory cytokines level with pretreatment with AVA in LPS-induced ALI.

## 5. Conclusion

**Figure 8:**
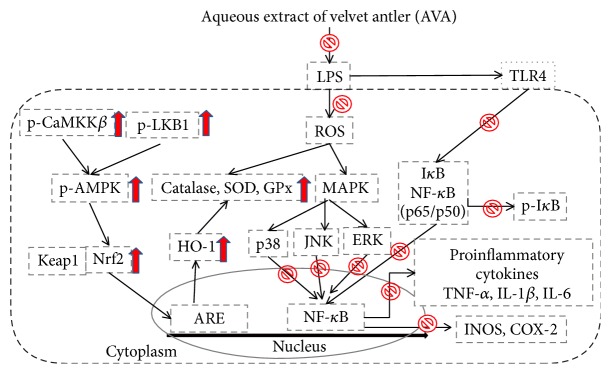
The schemes of the mechanism for the protective effect of AVA on LPS-induced inflammation.

In summary, pretreatment with AVA attenuated pulmonary histological changes and lung edema, reduced the inflammatory cell infiltration into lungs, and inhibited inflammatory cytokine production in the BALF. These data recommended that AVA has a particularly potent anti-inflammatory effect by suppressing the NF-*κ*B and MAPK activation and promoting AMPK/Nrf2 signaling pathways (Figure 8). The protective effect of AVA may be related to its ability to depress ROS generation, enhance antioxidant status, and regulate proinflammatory cytokine production. Thus, we believe that the present study could be used as a promising ingredient in functional foods or nutraceuticals against inflammatory diseases.
